# Embedding-based pair generation for contrastive representation learning in audio-visual surveillance data

**DOI:** 10.3389/frobt.2024.1490718

**Published:** 2025-01-13

**Authors:** Wei-Cheng Wang, Sander De Coninck, Sam Leroux, Pieter Simoens

**Affiliations:** IDLab, Ghent University—imec, Ghent, Belgium

**Keywords:** self-supervised learning, surveillance, audio-visual representation learning, contrastive learning, audio-visual event localization, anomaly detection, event search

## Abstract

Smart cities deploy various sensors such as microphones and RGB cameras to collect data to improve the safety and comfort of the citizens. As data annotation is expensive, self-supervised methods such as contrastive learning are used to learn audio-visual representations for downstream tasks. Focusing on surveillance data, we investigate two common limitations of audio-visual contrastive learning: false negatives and the minimal sufficient information bottleneck. Irregular, yet frequently recurring events can lead to a considerable number of false-negative pairs and disrupt the model’s training. To tackle this challenge, we propose a novel method for generating contrastive pairs based on the distance between embeddings of different modalities, rather than relying solely on temporal cues. The semantically synchronized pairs can then be used to ease the minimal sufficient information bottleneck along with the new loss function for multiple positives. We experimentally validate our approach on real-world data and show how the learnt representations can be used for different downstream tasks, including audio-visual event localization, anomaly detection, and event search. Our approach reaches similar performance as state-of-the-art modality- and task-specific approaches.

## 1 Introduction

Today, around 55 percent of the global population is living in an urban area or city, and this number is expected to rise to 68 percent by 2050 (United Nations Department of Economic and Social Affairs, 2018). To support this urbanization in a sustainable way, smart cities deploy a variety of sensor, networking and data analysis technologies to improve their operational efficiency and safety measures. Cameras and microphones are two prevalent sensors in smart city applications. Cameras primarily serve surveillance functions, facilitating crime prevention and traffic monitoring, while microphones are utilized for detecting phenomena such as gunshots or glass shattering (Mydlarz et al., 2017). Deploying cameras and microphones in the same location enables more comprehensive situational insights. Audio and video cues provide complementary information, which enhances the robustness of event detection against challenges encountered in real-world settings, including noise, occlusions, or low-light conditions (Bajovic et al., 2021).

Deep neural networks are currently the state-of-the-art solution for audio-visual surveillance tasks such as vehicle detection (Mao et al., 2020), violent scene detection (Ullah et al., 2023), and sound tagging (Bai et al., 2022). However, training these models requires large (labelled) datasets that are expensive to collect. Furthermore, research indicates the advantages of employing location-specific models for surveillance (Leroux et al., 2022), further increasing the amount of training data and associated labels that need to be collected.

The objective of this work is to design a scalable framework for learning representations of real-world audio-visual surveillance data in a self-supervised manner. The resulting representations should generalize well to a wide range of downstream surveillance tasks, meaning that the training of task-specific models that will take these representations as input requires little or no labelled data. Examples of downstream tasks for smart city surveillance include event localization (Ran et al., 2022), anomaly detection (Wu et al., 2022; Kumari and Saini, 2022; Leporowski et al., 2023) and event search (Munjal et al., 2019).

Self-supervised learning of transferable representations is typically achieved by training a feature extraction model on a pretext task. Contrastive learning, a specific type of self-supervised learning, formulates the training objective in terms of a distance metric between the representations of a pair of input samples. The goal is to minimize the distance for semantically similar instances (positive pairs) and maximize it for dissimilar instances (negative pairs). The process of generating positive and negative pairs during training is a crucial factor in obtaining transferable features. Negative pairs are often generated through random sampling from the dataset. Positive pairs can be constructed without requiring label information by pairing a sample with an augmented version of that sample. Such augmentations are straightforward in the case of static images, but much harder to design for temporal data (Qian et al., 2021). In the case of multi-modal data, positive pairs can be naturally formed by treating audio and video clips sampled at the same timestamp within a stream as positive pairs, a pair generation mechanisms known as Audio-Visual Synchronization (AVS) (Aytar et al., 2016). To distinguish it from our approach, we refer to it as Temporal-based Pair Generation (TPG) to highlight that typical Audio-Visual Synchronization takes temporal cues when generating data pairs.

**FIGURE 1:**
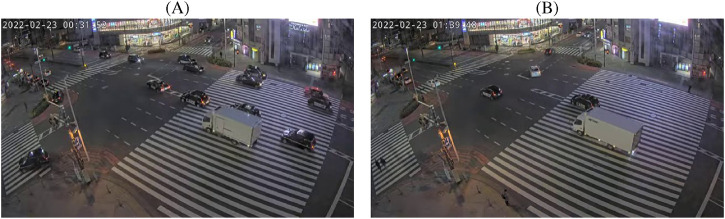
Sampling data from different timestamps may result in false negative pairs if both timestamps share a similar audio-visual context. **(A)** Visual frame at 00:31:52. **(B)** Visual frame at 01:39:40.

TPG however introduces two challenges related to the semantic repetition that is observed in audio-visual surveillance data over time. First, a large temporal distance between two fragments of a recording does not guarantee a semantical difference between these fragments. Ambulances, police cars, buses, auditory beacons for visually impaired pedestrians, or vans with similar appearance are only a handful examples of scenes recurring at unpredictable and variable intervals. One example taken from a surveillance camera in Tokyo is shown in Figures 1A, B shows two visually and aurally similar trucks appearing at different time frames. When sampling a data pair where the visual modality is taken from (A) and the audio modality from (B), this pair is labeled as negative based on the time stamps, despite their semantic similarity. Such mislabeled pairs, referred to as *false negatives*, compromise the training process and cause the learned embedding spaces to lose the semantic meaning (Zolfaghari et al., 2021; Sun et al., 2023; Chuang et al., 2020).

**FIGURE 2:**
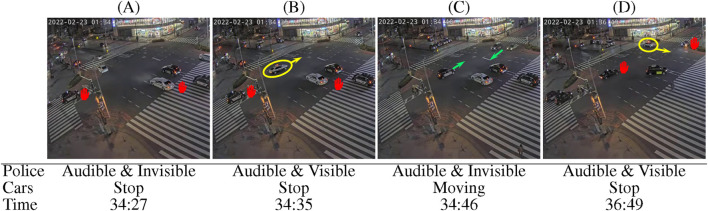
Example stills illustrating two instances of a police car passing an intersection with activated siren, forcing other vehicles to stop. The police car is indicated in yellow. Stopped and moving cars are indicated with red signs and green arrows respectively. **(A)** Only the siren of the police car is audible. **(B)** The police car enters the scene. **(C)** The police car leaves the scene but siren is still audible. **(D)** On a later time, a police car enters the scene with a different view angle.

Another limitation of relying on temporal cues to generate positive and negative pairs in contrastive learning arises from the information bottleneck in the training objective. Since all supervision information for learning a representation of one element comes from the contrasting element (Tian et al., 2020a), the representations are *minimal sufficient*, meaning that they are focused on the mutual information between the samples of positive pairs. While this is effective when the downstream task is aligned with the pretext task, the minimal sufficient may not contain enough information to generalize across multiple downstream tasks (Tsai et al., 2021; Wang et al., 2022; Feichtenhofer et al., 2021). Increasing the number of positive pairs for each sample can address this limitation, as it makes the pretext task more challenging and encourages the representations to encode richer information (Chuang et al., 2020; Khosla et al., 2020; Tian et al., 2020a). With TPG, each video clip forms a single positive pair with its corresponding audio, leading to minimal sufficient representations that only contain information on objects that are simultaneously audible and visible. However, real-world events are complex, involving multiple elements at different time intervals, as shown in Figure 2. Figures 2A–C shows the progressive stages of a police car cautiously passing by a busy intersection. Due to the relative distance and velocity between the camera and the vehicles, as well as interactions between vehicles, each timestamp is a unique combination of visual and audio cues of the same event. Because of the information bottleneck, the representation of this scene might not contain all the visual information (police car and vehicle stopping) with its audio modality (siren). In the case of Figure 2B, when learning a minimal sufficient representation, part of the visual information could be ignored (e.g., vehicle stopping), although this information could be essential for downstream tasks. For example, during an emergency, the audio of the siren and the visual cue of other vehicles stopping are crucial for managing the traffic light before the police car enters the camera’s view. Meanwhile, Figure 2D shows a similar event occuring at another time, where a police car approaches from a different road. By creating positive pairs using samples from both events, the learned representations will contain more comprehensive information, accommodating the complexities of real-world scenarios.

To reduce false negatives as well as to learn representations with sufficient information, semantically similar events should be mapped together in the embedding space. In this paper, we introduce the *Embedding-based Pair Generation* (*EPG*) mechanism as an alternative for TPG to sample positive or negative pairs. Our approach detects false negative pairs by calculating a distance between the embeddings of two instances of the *same* modality. Furthermore, we propose a new loss that considers multiple positives simultaneously to learn representations that contain more information, further improving the transferability of the learned features to a variety of downstream tasks. We train a pseudo-Siamese network to encode the audio segments and video frames to the same embedding space. After training, the model has learnt what audio typically corresponds to certain visual inputs and *vice versa*. The two deep neural networks can then serve as feature extractors, jointly or separately, for downstream tasks.

To summarize, our main contributions are as follows:

1. We identify the inherent flaws in applying the widely used audio-visual correspondence to smart surveillance data. An embedding-based pair generation is introduced to tackle this problem;
2. We study the limitation of minimal sufficient representation for audio-visual representation learning in surveillance. We then propose a novel loss to encode richer task-relevant information to improve the performance on downstream tasks;
3. We evaluate our approach with supervised downstream tasks and demonstrate the effectiveness of our improvement comparing to the-state-of-the-art approaches on audio-visual representation learning. We further qualitatively evaluate our approach on two unsupervised tasks applied on real-world surveillance data.

The remainder of this paper is structured as follows. Section 2 provides the related work of audio-visual representation learning, self-supervised learning, and how positives and negatives are generated for contrastive learning. In Section 3, we describe the mechanism of embedding-based pair selection and how we incorporate multiple positives in the contrastive loss. The downstream tasks and datasets used to evaluate the learnt representation are explained in Section 4. We then describe the implementation details and the discussion on the experimental results in Section 5. We conclude in Section 6 and give some directions for further research.

## 2 Related work

Our work lies at the intersection of three domains: audio-visual representation learning, self-supervised representation learning, and pair generation for contrastive learning. In the following subsections, we provide an overview of the approaches in each of these fields that are most pertinent to our work.

### 2.1 Audio-visual representation learning

The analysis of audio-visual data is gaining popularity as audio and visual information offer complementary insights on the same content. The two modalities are expected to align, either at the frame level or the instance level. Jointly considering both modalities benefits the analysis of audio-visual tasks such as active speaker detection (Afouras et al., 2020b), sound source localization (Zhou et al., 2023), lip reading (Afouras et al., 2020a), or video forensics (Feng et al., 2023).

To encode audio-visual representation, most frameworks employ three components: an audio encoder, a visual encoder, and a projector. This modular design accounts for the distinct characteristics of audio and visual data, requiring different processing configurations, such as varying network architectures and learning schedules. Once high-level information is extracted by the encoders, the embeddings are connected by passing them through the projector. This setup is especially useful for tasks like anomaly event recognition (Gao et al., 2024), where labelled data is easier to obtain, allowing for straightforward end-to-end training to learn audio-visual representations.

However, in most real-world scenarios, audio-visual data is collected continuously, making it challenging to obtain annotations. To address this, self-supervised learning (SSL) is a promising approach to leverage the semantic synchronization between audio and video. The inherent correlation between audio and visual elements serves as a natural indicator when designing the pretext task in self-supervised learning. When applying SSL to audio-visual data, the general concept involves training a model to differentiate between matching and non-matching pairs of video segments and audio excerpts. Negative pairs are constructed either by sampling audio and video from different recordings, known as audio-visual correspondence (AVC), or by sampling from different offsets in the same recording, termed audio-visual synchronization (AVS).

AVC as pretext task was first introduced by Arandjelovic and Zisserman (2017), who demonstrated that the learnt representations obtained competitive results on both audio tasks, such as sound classification, and visual tasks, such as image classification and object detection. Subsequent works extended AVC to tasks such as action recognition (Morgado et al., 2021), active speaker detection (Afouras et al., 2020b) or sound source localization (Zhou et al., 2023). More recently, Huang et al. (2024) proposed a hybrid approach combining generative SSL objectives with contrastive learning. With a joint loss function, both inter-modal and intra-modal relationships can be considered by the model.

AVS, as a more nuanced pretext task, leverages the temporal synchronization between audio and video to pretrain the model. This approach has been successfully applied to tasks such as lip reading (Afouras et al., 2020a), video forensics (Feng et al., 2023) and active speaker detection (Wuerkaixi et al., 2022).

### 2.2 Self-supervised representation learning

While supervised learning has made a great achievement in many research domains, accessing reliable annotations for data is sometimes expensive or impractical. Self-supervised learning aims to learn a representative embedding by leveraging the information within the data instead of relying on the supervision of annotations. SSL introduces pretext tasks, which are auxiliary tasks designed to train the model to learn representations that can later be applied to downstream tasks. These pretext tasks may not directly relate to the target task but serve as an effective means of extracting generalizable embeddings.

According to the type of the pretext task, SSL approaches can be divided into three different categories (Wang Y. et al., 2022): generative, predictive and contrastive. Below, we briefly describe all three and will then focus on the contrastive approaches as these form the basis for our work.

Generative SSL employs generative models, such as AutoEncoders (AEs) or Generative Adversarial Networks (GANs), coupled with pixel-level reconstruction loss functions to learn representative features. This approach is particularly popular in the field of computer vision (He et al., 2022; Wu et al., 2022; Wu et al., 2023). While pixel-level reconstruction is an intuitive and effective pretext task, the generative SSL methods can be hard to train. During the training of the generative model, model tend to focus overly on background details at the expense of the foreground content. This happens particularly when the foreground content is relatively small in terms of frame ratio, known as the foreground-background imbalance. Another common challenge is the object scale imbalance, where the size of the objects varies when the camera has a more oblique view, as discussed in (Sampath et al., 2021). Both problems require additional mechanisms to focus on specific semantic information.

Predictive SSL methods utilize self-generated labels derived from predefined transformations of the input data to guide network training. Pretext tasks such as classifying rotated versions of the original image (Gidaris et al., 2018), or arranging image regions within a jigsaw puzzle (Misra and Maaten, 2020) have been demonstrated to result in high-level features of images. These pretext tasks preserve the semantic meaning of the content. It is however not trivial to design good pretext tasks for temporal data, e.g., in surveillance applications, due to the added complexity of sequence dynamics.

Finally, contrastive SSL aims to overcome the challenges encountered in the aforementioned approaches. Contrastive SSL compares pairs of data samples to learn representations that maximize similarity for positive pairs (semantically similar samples) and minimize similarity for negative pairs (semantically dissimilar samples). Positive pairs typically consist of different augmentations of the same input sample (Chen et al., 2020; Zbontar et al., 2021), while negative pairs are created by randomly pairing samples from the dataset. Both inputs are projected to a shared embedding space. By training the model to maximize the mutual information between embeddings of samples in positive pairs and minimize that of samples in negative pairs, the model learns to extract high-level features that can be used for downstream tasks. Contrastive SSL extends to multimodal data by forming pairs with samples from each modality. For instance, in audiovisual representation learning, video fragments paired with corresponding audio fragments represent positive pairs, while combinations of video frames and randomly selected audio snippets serve as negative pairs (Sun et al., 2023; Ran et al., 2022). Text is another modality commonly used in conjunction with video, in particular to learn language-video representations by pairing the video with its caption (Zolfaghari et al., 2021; Zhang et al., 2023).

While contrastive learning has been proven to be effective in many applications, there are two notable limitations, namely, *false negatives* (Sun et al., 2023) and the minimal sufficient representation (Tian et al., 2020a) problem. False negatives occur when semantically similar pairs are mistakenly labelled as negative due to the design of the pretext task. Zolfaghari et al. (2021) describes the impact of false negatives and proposes identifying influential samples. These samples, which are more likely to be false negative samples, have high feature similarity with other samples and should be removed. Similarly, Sun et al. (2023) proposes a statistical approach to locate false negatives by considering the similarity between the same modality of different sample pairs. The information bottleneck leading to minimal sufficient representation, containing information that is sufficient for the pretext task, is rather rare in scholarly discussions. Tian et al. (2020a) thoroughly describes the concept with theoretical and empirical proof, stating that when the downstream tasks are not aligned with the pretext task, it might downgrade the representative of learnt features. This issue is especially pronounced when applying contrastive learning to surveillance data, where events unfold over time and involve multiple stages. A minimal sufficient representation may not be able to describe the different stages of an event. To improve the usability of learned representations in downstream tasks on such data, several works aim to incorporate multiple positives in the objective function (Chuang et al., 2020; Khosla et al., 2020; Tian et al., 2020a). By considering multiple semantically related positives in the objective function, models can better capture the diverse aspects, improving the richness and generality of the learnt features.

### 2.3 Pair generation for contrastive learning

The selection of positive and negative pairs is a crucial factor in contrastive learning (He et al., 2020; Shah et al., 2022). Many works assume that randomly selected inputs lack semantic similarity and can be used as negative pairs. However, this assumption can introduce false negative pairs. This issue, a phenomenon also referred to as *sampling bias*, has been shown to hinder performance. Chuang et al. (2020) empirically demonstrate significant performance gains across multiple research domains when false negatives are avoided. Other recent studies prove theoretically and empirically that the quality of the negative samples is more important than their quantity (Kalantidis et al., 2020; Zhu et al., 2021).

Recent works have explored several strategies to refine the process of generating positive and negative data pairs. At the level of instance sampling, Kalantidis et al. (2020) enhance training efficiency and the quality of the learned representations by synthesizing hard negatives. These hard negatives closely resemble positive pairs and are therefore challenging for the model to distinguish. During the training process, novel hard negatives are synthesized as feature-level linear combinations of the currently hardest examples. Tian et al. (2020b) focus on enhancing the diversity of the sampled positive pairs. They argue that improving the diversity in positive pairs helps the model learn representations that are invariant to nuisance variables, since the representations are focused on the mutual information across all positive views. Zhu et al. (2021) introduce a feature transformation technique that manipulates features to create both hard positives and diverse negatives. Beyond improving the pair selection, Chuang et al. (2020) propose the *debiased contrastive loss*, a novel training objective function that considers the approximated distribution of negative samples instead of relying on explicit negative samples.

## 3 Proposed method

In the following sections, we will first explain the different components of the framework. Then, we introduce the novel embedding-based pair generation (*EPG*) mechanism designed to reduce the number of false negatives. Finally, we introduce a novel loss function and elaborate on how this loss function might address the challenge of minimal sufficient representations.

### 3.1 Architecture

**FIGURE 3:**
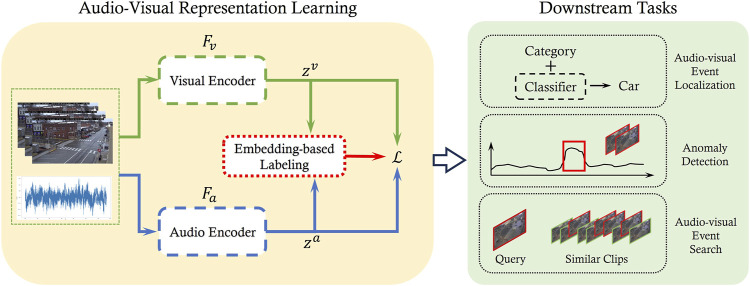
Overview of the framework. A pair of video clip $x^{v}$ and audio segment $x^{a}$ are served as the input of a two stream pseudo-Siamese network. The network consists of a visual encoder $F_{v}$ and an audio encoder $F_{a}$ to project the input into the same embedding space. A embedding-based label of the data pair is calculated to determine whether the data pair is positive or negative. These labels are later used to compute the proposed loss and to update the network. After the pseudo-Siamese network is trained, both encoders can be used as a feature extractor jointly or separately for downstream tasks.

As illustrated in Figure 3, the audio-visual representation learning block follows a pseudo-Siamese structure with two encoders: $F_{v}$ and $F_{a}$ . Both encoders are deep convolutional networks, designed to process visual and audio information, respectively. Where in conventional Siamese architectures, the parameters are shared between the encoders, here the encoders have a different structure, hence the name *pseudo*-Siamese.

The encoders project the audio and video onto a shared embedding space Z . Surveillance data X is first split into short clips, where each clip x comprises the sequence of frames $x^{v}$ and an audio segment $x^{a}$ . By selecting from the frames and segments of X , we first generate a data pair $p_{m,n}=\left(x_{m}^{v},x_{n}^{a}\right)$ , consisting of the m -th video segment and the n -th audio fragment. With $F_{v}$ and $F_{a}$ , $p_{m,n}$ is encoded into Z , yielding $\left(F_{v}\left(x_{m}^{v}\right),F_{a}\left(x_{n}^{a}\right)\right)=\left(z_{m}^{v},z_{n}^{a}\right)$ . By utilizing $\left(F_{v}\left(x^{v}\right),F_{a}\left(x^{a}\right)\right)$ and the pair generation mechanism, a contrastive loss L is calculated to train the network. Once $F_{v}$ and $F_{a}$ are trained, the encoders can be used as feature extractors for downstream tasks, either jointly or independently.

### 3.2 Embedding-based pair generation

Recognizing the limitations of relying solely on time offsets to ascertain semantic dissimilarity, we introduce an alternative solution to identify temporally non-aligned but semantically similar data pairs with the distance in the embedding space.

The set of possible pairs is denoted as $P=\left\{p_{m,n}=\left(x_{m}^{v},x_{n}^{a}\right)\right\}$ . Instead of solely relying on the condition m=n to label the pair $p_{m,n}$ pair as positive, we propose to calculate the mutual information between $x_{m}$ and $x_{n}$ in the embedding space Z . The mutual information $I\left(z_{m},z_{n}\right)$ can be computed using a distance metric $d_{Z}\left(\cdot \right)$ such as Euclidean distance or cosine similarity (Boudiaf et al., 2020). If the mutual information between the video or audio embeddings, $I\left(z_{m}^{v},z_{n}^{v}\right)$ or $I\left(z_{m}^{a},z_{n}^{a}\right)$ , is higher than a threshold δ , we say $x_{m}$ and $x_{n}$ are semantically similar, even though they are recorded at different times.

To identify whether the two elements of the pair $p_{m,n}$ are semantically similar, we calculate the label $y^{e}\left(m,n\right)$ with the following equation:

$$y^{e}\left(m,n\right)=\left\{\begin{array}{cc} 1, & d_{Z}\left(x_{m}^{v},x_{n}^{v}\right)\leq \delta \vee d_{Z}\left(x_{m}^{a},x_{n}^{a}\right)\leq \delta \\ 0, & d_{Z}\left(x_{m}^{v},x_{n}^{v}\right)>\delta \wedge d_{Z}\left(x_{m}^{a},x_{n}^{a}\right)>\delta \end{array}\right.$$ (1)

Intuitively, this definition ascertains that when two audio fragments are semantically similar $F_{a}\left(x_{m}^{a}\right)\approx F_{a}\left(x_{n}^{a}\right)$ , we assume that the corresponding video fragments in time also should be semantically similar, and *vice versa*. Conversely, two video fragments close in embedding space are hypothesized to have semantically close audio fragments. Hence, positive pairs can be constructed by mixing modalities of $x_{m}$ and $x_{n}$ . With mutual information between the video or audio embeddings, we can further identify the false negative pairs and consider them as positive. Consequently, these positive pairs can also be used to reduce the limitation of minimal sufficient representation.

### 3.3 Contrastive loss with multi-positive pairs

Given that the encoders of both modalities are trained to predict similar feature representations for temporally aligned audio and video clips, they tend to focus on information present in both modalities. Contrastive loss guides the training of the encoders such that the video and audio embedding are close: $F_{v}\left(x_{m}^{v}\right)\approx F_{a}\left(x_{n}^{a}\right)|m=n$ . This means that $F_{v}\left(x_{m}^{v}\right)$ learns to eliminate all information not present in $x_{m}^{a}$ , and *vice versa*. As explained in the introduction, other audio segments may contain complementary information, but this will be eliminated in the representation of $F_{v}\left(x_{m}^{v}\right)$ .

The main purpose of including multiple modalities however, is to complement each other, providing additional information when an object or person can not be observed in one of the modalities. This is particularly a problem for data pair generation that only relies on temporal alignment since it allows only one positive pair for each time frame:

$$\forall x_{n}^{v}\in X,\exists !x_{m}^{a}|m=n.$$ (2)

To address this limit of minimal sufficient representation, we propose a modification to the conventional contrastive loss function that uses multiple positive pairs identified through embedding-based distance Equation 1.

Different from temporal-based pair generation, the embedding-based pair generation mechanism does not solely rely on temporal information to create positive pairs with $x_{m}^{v}$ . With the semantic similarity of the embedding space, the limitation due to Equation 2 may be reduced as there can be multiple positives for $x_{m}^{v}$ :

$$|\left\{x_{n}^{a}\in X|y^{e}\left(m,n\right)=1\right\}|\geq 1$$ (3)

The upper bound on the mutual information between $x_{m}^{v}$ and the union of all elements in the set in 3 is higher than the mutual information between $x_{m}^{v}$ and $x_{m}^{a}$ . As a result, including multiple positives will likely retain more information on $x_{m}^{v}$ in the representation $F_{v}\left(x_{m}^{v}\right)$ , which will benefit downstream task performance. Inspired by the loss function proposed in (Hadsell et al., 2006), we proposed a modified loss function to fit the multi-positive found by Equation 3.

$$\begin{array}{cc} L^{\mathrm{EPG}}\left(X\right)=\underset{x_{m}^{v}\in X}{\sum } & \underset{x_{n}^{a}\in X}{\sum }\left(y^{e}\left(m,n\right)\cdot d_{Z}{\left(x_{m}^{v},x_{n}^{a}\right)}^{2}+\right.\\ & \left(1-y^{e}\left(m,n\right)\cdot max{\left(\tau -d_{Z}\left(x_{m}^{v},x_{n}^{a}\right),0\right)}^{2}\right). \end{array}$$ (4)

The distance metric $d_{Z}\left(x_{m}^{v},x_{n}^{a}\right)$ measures the distance between $x_{m}^{v}$ and $x_{n}^{a}$ in the shared space Z , which is bounded by a predefined constant τ in the second term of Equation 4. Note that, due to the symmetry between each modality of two semantically similar data pairs, Equation 4 is equivalent to pairing each visual modality $\left(x_{m}^{v}\right)$ within the dataset with each audio $\left(x_{n}^{a}\right)$ . The distance function $d_{Z}$ can represent any similarity metric. In this paper, we obtained the best results using a weighted combination of the Euclidean distance ‖⋅‖ and the cosine similarity $S_{c}\left(\cdot \right)$ . As cosine similarity yields larger values for higher similarity; the distance function $d_{Z}\left(\cdot \right)$ is defined as follows:

$$d_{Z}\left(x_{m}^{v},x_{n}^{a}\right)=\omega \cdot \|z_{m}^{v}-z_{n}^{a}{\|}^{2}+\left(1-\omega \right)\cdot \left(2-\left(S_{c}\left(z_{m}^{v},z_{n}^{a}\right)+1\right)\right),$$ (5)

where ω is a value between 0 and 1 to control the ratio between the two distance functions. The use of ω is discussed in Section 5.4.

## 4 Experimental setup

In this section, we describe the experiments conducted to evaluate the quality of the learned representations across various downstream tasks relevant to smart surveillance applications. The evaluation includes one supervised downstream task, namely, audio-visual event detection, and two unsupervised tasks: anomaly detection and event query. For all tasks, the pseudo-Siamese network is first pretrained on the audio-visual data using the objective function of Equation 4 in a self-supervised manner. Subsequently, the weights of the audio and/or visual encoders were frozen and considered as fixed feature extractors while training a small network for each of the downstream tasks.

### 4.1 Implementation details

For all experiments, we maintain consistent configurations for data preprocessing, network architecture, and training procedure. Task-specific variations are described in subsequent sections. The audiovisual dataset is first segmented into 1-s, non-overlapping recordings, each containing synchronized audio segments and video frames.

Video recordings are downsampled to 5 fps, and each frame is resized to 398×224 pixels. To align with the visual encoder’s input specifications, each frame is further divided into two overlapping 224×224 crops. These two crops are treated as independent frames that map to the same audio segment, and are then jointly considered during the inference phase.

Audio segments are resampled to 44,100 Hz and transformed into log-mel spectrograms, following the configuration outlined by Adapa (2019). This transformation utilizes a window size of 256, a hop length of 694, and a total of 128 bins.

The framework consists of a pseudo-Siamese network with a visual encoder $F_{v}$ and an audio encoder $F_{a}$ . $F_{v}$ is built based on X3D-M (Feichtenhofer, 2020), featuring one convolutional layer, four residual blocks, and a final classification layer. We take the implementation from PyTorchVideo but replace its last layer with a fully-connected layer with 512 neurons to align with the dimensions of the audio representations. $F_{a}$ is implemented as a ResNet18 model, taking the log-mel coefficients as input. The parameter counts for $F_{v}$ and $F_{a}$ are 4.02 million and 4.16 million, respectively. Training specifics differ: $F_{v}$ trains with a learning rate of 2e−4 and a decay of 1e−5 , while $F_{a}$ is trained with a learning rate of 1e−3 and a decay of 1e−5 .

For $F_{v}$ , we take the pre-trained weights of X3D-M, which is trained on Kinetics-400, provided by PyTorchVideo. As for $F_{a}$ , we train it from scratch by freezing the pre-trained $F_{v}$ and exclusively training $F_{a}$ . Subsequently, using a layer-wise learning approach (Belilovsky et al., 2019), both encoders undergo iterative training.

The embedding space Z is designed to capture semantically meaningful information, which may not be guaranteed when training from scratch. To ensure robust initialization, we only consider a pair as positive when the two modalities are sampled from the same timestamps during the first epoch of training. In all subsequent epochs, training shifts to the embedding-based label $y^{e}\left(m,n\right)$ , guided by the loss function L . Throughout training, the weight parameter ω for the distance function in Equation 5 is kept constant at 2.5, and σ is set to 0. An ablation study of these parameters is provided in 5.4. As δ is the threshold to determine whether the distance in embedding space of a temporally misaligned data pair is smaller than a temporally aligned pair, we set the δ as the distance between the embeddings of the temporally aligned pair $\left(\delta =d_{Z}\left(x_{m}^{v},x_{m}^{a}\right)\right)$ . Thus, the threshold δ adapts dynamically during training based on the embedding space.

### 4.2 Supervised tasks

After pretraining the audio and video encoders, they can be used as feature extractors for downstream tasks. The first task we consider is event localization, which aims to pinpoint specific predefined events within an audiovisual stream, such as the entry of a car. This task can be cast as a supervised learning problem by following the protocol outlined in (Ran et al., 2022). Long recordings are divided into short clips, and the objective is to perform binary classification to determine whether a given clip contains the target event.

#### 4.2.1 Dataset

Finding real-world surveillance datasets that contain video, audio and labels is challenging. Some established datasets, such as those used in Benfold and Reid (2011); Ristani et al. (2016), have been taken down due to privacy concerns. Other datasets primarily consist of very short clips gathered from diverse locations, often sourced from video-sharing platforms like YouTube (Aytar et al., 2016; Perez et al., 2019; Danesh Pazho et al., 2023). While these datasets suffice for certain supervised tasks, such as violence detection, they fall short for our purpose of learning features in a semi-supervised manner over long audiovisual streams.

**FIGURE 4:**
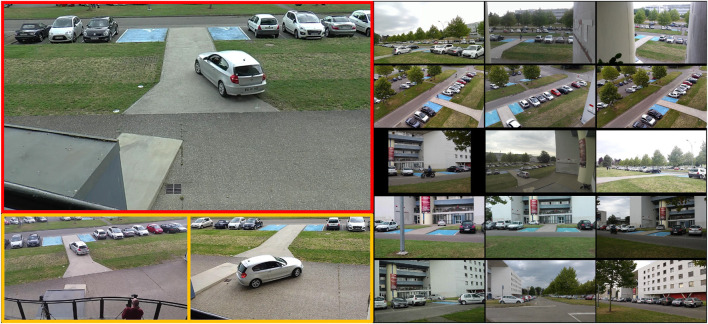
Camera setup for ToCaDa Scenario 1. The camera view used for training is marked in red, while the camera views that are used for similar are labelled as yellow. The rest of the camera views are used as challenging . Note that both similar sets and challenging sets are used for testing.

We decided to use the Toulouse Campus Surveillance Dataset (ToCaDa) (Malon et al., 2020) to validate our approach. The ToCaDa dataset encompasses two distinct scenarios, each captured by multiple cameras strategically positioned to record simultaneously audio and video. Some cameras have overlapping fields of view. The events in the videos are scripted to demonstrate a possible burglary involving 20 actors playing roles as pedestrians or suspects. Each video has an approximate duration of 300 s and comes with detailed annotations for both audio and video events. Most videos in this dataset contain only a limited number of events, making a meaningful split of each video across train and test set difficult. However, scenario one of the ToCaDa dataset contains a higher number of cameras observing the same scene, including some with slightly different viewpoints, see Figure 4. We therefore use the footage of Camera 2 as the training set for learning representations, and evaluate event localization on the audiovisual recordings from all other cameras.

#### 4.2.2 Evaluation procedure

We evaluate the transferability of the learned representations to this supervised classification task by adopting the linear evaluation protocol from Wang et al. (2022). After pretraining, the weights of the feature extractors are frozen and a one-layer linear classifier is trained using cross-entropy loss as the objective function. After training the classifier, we evaluate performance using a segment-wise classification accuracy matrix, again following the protocol presented in (Ran et al., 2022).

#### 4.2.3 Baseline methods

We first evaluate our method by comparing it with existing audio-visual representation learning methods, as well as the *TPG* baseline. Additionally, since the data contains both audio and video, we compare the classification results of our method with visual-only and audio-only methods.

For multi-modal audio-visual representation learning, we compare our method with TACMA and MAVil. TACMA (Ran et al., 2022) is a self-supervised representation learning technique specifically designed for audio-visual event localization. TACMA employs a Barlow-Twins architecture to learn representations and includes a cross-modal attention module to enhance audio-visual information capture. However, because the cross-modal attention module is trained in a supervised manner, we exclude it and use only the AV-BT module to generate the representations. MAViL (Huang et al., 2024) is a recent method that has demonstrated strong performance in event classification tasks across general audio-visual datasets.

Both TACMA and MAViL are designed for datasets containing short videos with diverse content and scenarios. To ensure compatability with their training scheme, we segment the long ToCaDa training videos into 10-s, non-overlapping subclips. Unlike TACMA, MAViL relies on negative pairs to compute inter-modal contrastive loss. To enable this, we pair the audio and video from different subclips to create negative pairs for MAViL.

For the *TPG* baseline, we use the same training procedure as our method, with the only difference being the pair generation strategy.

For visual-only benchmarks, we employ the EfficientNet (Tan and Le, 2019) and X3D (Feichtenhofer, 2020) models. EfficientNet is pretrained on the ImageNet dataset, and we select the EfficientNet-B0 variant, which has 4.03 million parameters, making it comparable in scale to the $F_{v}$ encoder in our method. For X3D, our choice is the X3D-M variant pretrained on Kinetics-400, which contains 3.76 million parameters. Both models and their pretrained weights are sourced from the PyTorch and PyTorchVideo repositories. To use these models as baselines, we remove their final classification layers and use the outputs of the remaining pretrained network as representations.

TACMA and X3D-M process input frames at a resolution of 256×256 , while EfficientNet-b0 operates at 224×224 . For fair comparison, we first downsize all frames to 224times224 and then upsample them to 256×256 for use with TACMA and X3D-M.

For all other configurations, we follow the original preprocessing steps specified in the respective works, except for MAViL. Since the authors of MAViL did not release their code or the pretrained model, we follow the implementation details and the pretrained model of the reproduction reported in (Tseng et al., 2024).

For the audio-only benchmarks, we use the best-performing model from the DCASE19 urban sound tagging task (Adapa, 2019). The official implementation and pretrained weights were obtained from the author’s GitHub repository^1^. Similarly to the visual-only benchmarks, we remove the classification layer from the pretrained network and use its output as feature representation for downstream evaluation.

### 4.3 Unsupervised tasks

We also evaluate our framework in two unsupervised tasks commonly used in surveillance: anomaly detection and query-guided event search. The task of anomaly detection involves identifying inputs that deviate from normal behavior. Since the behaviors of interest are not defined beforehand, anomaly detection is a challenging task that requires high-quality input features to discern subtle deviations. The query-guided event search task is to locate events in a video similar to a given query event. For instance, if the query is a clip containing a joyriding car with distinct audio or visual characteristics, the task is to identify other timestamps in the recording where similar events occur.

#### 4.3.1 Dataset

The ToCaDa dataset, while valuable for tasks like event localization, is less suited for anomaly detection and query-guided event search due to its limited video length and restricted diversity of actions occuring. Similarly, widely-used datasets with annotated anomalies, such as Avenue (Lu et al., 2013) and ShangHaiTech (Liu et al., 2018), contain only visual cues. As an alternative, we collected real-world audio-visual surveillance footage from a publicly available live stream on YouTube^2^,^3^. This audiovisual stream captures a main intersection in Tokyo’s Shinjuku district, observed from a a high vantage point, providing a representative setting for urban surveillance. Some stills from the recordings are shown in Figure 1, showcasing different types of vehicles, bikes and pedestrians with their accompanying sounds. For in-depth evaluation, we recorded four 4-hour-long videos from two different dates under different lighting conditions. The videos are recorded during two time windows: 15:00 to 19:00 (daytime) and 19:00 to 23:00 (nighttime), on a Tuesday and a Thursday.

#### 4.3.2 Evaluation procedure

Since the Tokyo dataset is not annotated, we conduct a qualitative evaluation between different methods of anomaly detection and query-guided event search.

The anomaly score for a clip $x_{m}=\left(x_{m}^{v},x_{m}^{a}\right)$ is calculated using the distance function of Equation 5. A clip is flagged as anomalous if the score is higher than a threshold. For each 4-hour-long video, a separate model is trained to learn audio-visual representations. The threshold for anomaly detection is dynamically adapted for each video and set to μ+2σ where μ and σ represent the mean and standard deviation of the anomaly score on the training set. This threshold considers 95.45% of the training data as normal.

For query-guided event search, separate models are trained on each video to extract features. Query events have been selected manually. Events were searched in all recordings, but events within a window of 1 min before and after the selected event are excluded as search results. We rank the search results based on the distance between the query and the results in the embedding space.

#### 4.3.3 Baseline methods

For the anomaly detection task, we compare our method with five other approaches: two multi-modal fusion models (Kumari and Saini, 2022) (*Fusion*) and (Huang et al., 2024) (MAViL), one vision-only approach based on the X3D-M model, one audio-only baseline Adapa (Adapa, 2019) and the multi-modal *TPG* baseline.

As for the event search task, apart from the multi-modal *TPG* and MAViL (Huang et al., 2024) baselines, we compare our approach to a baseline that involves a straightforward fusion approach in which video and audio features of separately trained encoders are concatenated. Specifically, we concatenate visual features from X3D-M (Feichtenhofer, 2020) with audio features from Adapa (Adapa, 2019). We refer to this baseline as (*A + V*).

## 5 Experimental results

### 5.1 Audio-visual event localization

The experimental results for audio-visual event localization are summarized in Table 1, which reports the accuracy score for each method. The ToCaDa dataset consists of videos recorded from 18 different cameras. We trained on data from camera 2 and tested on all other cameras. To better assess the robustness of the learned features, we categorize the test cameras into two groups: (1) cameras with a view similar to the training camera (labeled as “similar”), and (2) cameras with distinct perspectives compared to the training camera (labeled as “challenging”). By reporting results separately, we ensure that the evaluation reflects the model’s generalization ability without artificially inflating accuracy due to overlapping cameras.

**TABLE 1:** Audio-visual event localization results on two subsets of ToCaDa dataset.

	Method	# Params.(M)	Similar	Challenging
Audio-Visual	TACMA (Ran et al., 2022)	150.46	77.41	67.20
	MAViL (Huang et al., 2024)	185.66	72.69	62.34
	*TPG*	15.2	71.33	60.47
	*EPG* (Ours)	15.2	86.92	77.48
Visual	EfficientNet^a^	4.01	85.65	73.20
	X3D-M^a^	2.97	90.21	71.84
	TACMA (Ran et al., 2022)	52.87	62.15	43.60
	MAViL (Huang et al., 2024)	85.74	65.23	51.44
	*TPG*	4.02	60.71	44.92
	*EPG* (Ours)	4.02	85.42	70.02
Audio	Adapa^a^ (Adapa, 2019)	4.16	85.22	82.47
	TACMA (Ran et al., 2022)	97.58	76.43	65.61
	MAViL (Huang et al., 2024)	85.74	77.24	62.30
	*TPG*	4.16	70.66	60.43
	*EPG* (Ours)	4.16	81.37	79.48

^a^ Denotes the model is pretrained on a large-scale dataset without any fine-tuning. The numbers are segment-wise classification accuracy following the protocol in TACMA (Ran et al., 2022).

The results highlight a significant performance gap between the two camera sets, with all methods achieving considerably higher accuracy on the “similar” set. These results corroborate findings in earlier research on the advantages of using location-specific methods for analyzing surveillance data (Leroux et al., 2022).

When comparing our approach with the other audio-visual techniques TACMA and MAViL, our approach demonstrates a substantial performance advantage. This difference can likely be attributed to the fact that these models are designed for more general purposes that require training on large-scale datasets with diverse content. Due to the more constrained nature of the ToCaDa dataset, these models struggle to learn sufficiently representative embeddings for event localization tasks. Examining the performance difference between TACMA and MAViL, we observe that while MAViL achieves great performance in general representation learning, its reliance on both reconstruction and contrastive loss with negative pairs defined based on temporal alignment, pose limitations in this dataset. In contrast, TACMA employs a Barlow-Twins architecture, which avoids the need for negative pairs, and only consider limited positive pairs.

Notably, when our model architecture is trained using the *TPG* pair generation strategy instead of the *EPG* strategy, a significant drop in accuracy is observed. This results highlights the advantages of accounting for semantic similarity in pair selection.

We also compare the classification results obtained using the video representations learned by our approach, TACMA and MAViL, with those from video-only based models. Our approach performs similar to the EfficientNet model, which was pretrained on the large scale ImageNet dataset. The best performance is achieved by the pretrained X3D-M model, which is expected given its architecture’s specific design for capturing motion information. In contrast, the self-supervised methods TACMA, MAViL, and *TPG* perform poorly in this task. MAViL’s weaker performance can be attributed to its reliance on reconstruction loss, which is susceptible to foreground-background imbalance. This imbalance makes MAViL more sensitive to differences in the scene. These results demonstrate that the feature representations obtained from our multimodal approach also transfer effectively to purely visual tasks.

For audio-only event localization, we observe a smaller drop in accuracy between the “similar” and “challenging” locations compared to video-only localization. This underscores the robustness of audio data, which is less sensitive to the exact positioning of sensors. The best results in this setting are obtained by the pretrained Adapa model. While our model performs slightly worse than Adapa, it achieves this without the need for pretraining on extensive labeled data. Furthermore, our proposed *EPG* approach consistently outperforms the *TPG* baseline, reinforcing the effectiveness of embedding-based pair selection in enhancing the quality of learned representations.

### 5.2 Anomaly detection

**FIGURE 5:**
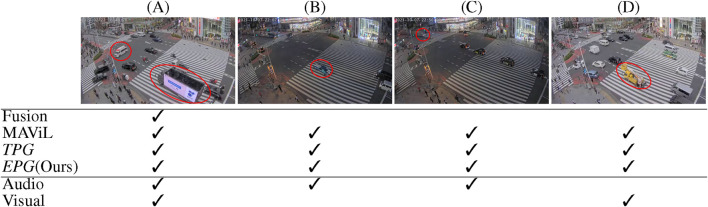
Examples of anomalous events. Check marks indicate which models flagged this event as anomalous. **(A)** An advertising truck waits at the crossroad while an ambulance passes with its siren wailing. **(B)** A sports car speeds by with the engine roaring. **(C)** An ambulance enters from the upper left corner. **(D)** A forklift passes through without any distinct noise.

Since the Tokyo dataset lacks annotations, we perform only a qualitative evaluation of anomaly detection performance. Figure 5 presents examples of anomalous events and indicates which methods were able to detect them. The shown events represent the four semantically distinct events with the highest anomaly score. These examples cover events with distinctive sounds, unique visual appearance, or a combination of both. This diversity demonstrates that the learned embedding space is semantically meaningful and contains information to identify various anomaly events.

In example (A), an advertising truck waits at the crossroad while an ambulance with wailing siren passes by. This anomaly is clearly identifiable through both visual and audio data. In example (B), a sports car speeds by with the engine roaring. While the sound of the sports car is highly distinctive, the car’s visual appearance is not particularly notable. The Fusion baseline (Kumari and Saini, 2022), which relies on multimodal data, fails to flag this event as an anomaly. In example (C), an ambulance arrives from the topleft corner with sirens on, then turns right and exits in the bottom left corner. In this case, the ambulance is not visually prominent but is clearly audible. Example (D) shows a yellow forklift passing through the intersection. There is no distinctive engine sound, leading audio-only methods to miss this anomaly.

These experiments show that by mapping audio and visual to the same embedding space, we can learn representations that effectively integrate information from both modalities. This enhances the ability to detect anomalies that are challenging to identify using a single modality.

### 5.3 Event search

**FIGURE 6:**
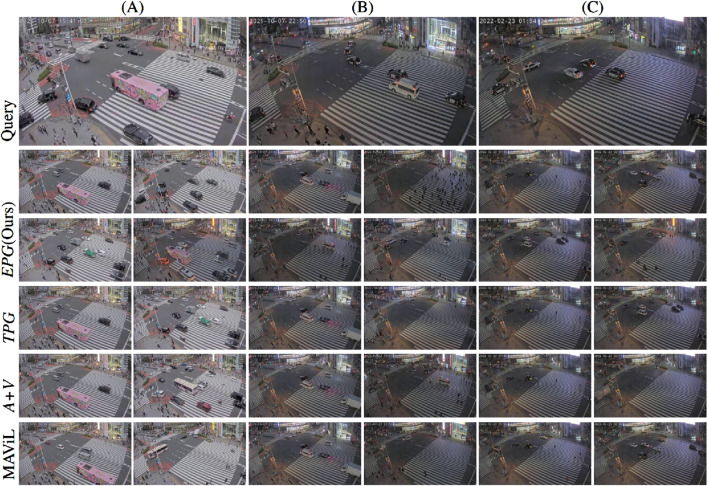
Examples of event query. The top row shows the query video, while the search results are shown in the 2–6 rows. The results of each methods are shown in a decreasing order from left to right and top to down. **(A)** A pink bus. **(B)** An ambulance with sirens on. **(C)** A police car with broadcast and siren on. We show the top 4 search results obtained with our method for each query events. For the other three methods, only the top two search results are reported in order not to overload the figure.

In our last set of experiments, we present examples of event search on the Tokyo dataset, as illustrated in Figure 6. The top row shows still frames of the (manually selected) query clips, while the following rows showcase the most related events identified by each method, shown in increasing order of embedding distance. We excluded a 1-min window before and after each query event from being searched. Similarly, for each method, we show only search results that are at least 1 minute before or after higher ranked search results.

Example (A) shows a pink bus entering the scene while the sound of a passing train can be heard in the background, though the bus itself produces no distinctive sound. All methods successfully locate a similar event where the same pink bus appears at a different time, again accompanied by the sound of a train in the background. Notably, the overlap between the bus’s visual presence and the train’s audio is brief. While the other approaches prioritize frames with visual similarity over audio similarity, MAViL selects a frame emphasizing the distinct train sound. In contrast, our method shows only a 1% difference in preference between audio-similar and visual-similar frames. Moreover, our approach identifies an additional instance of the same bus later in the surveillance stream, this time without a train in the background. Other detections from all methods are quite diverse but typically contain either a bus, multiple black cars or the sound of a train in the background.

In example (B), an ambulance enters with its siren wailing accompanied by a broadcast announcement. Besides the passage in the query clip, the ambulance appears at least six more times during the night. All methods detect one particular instance where the ambulance enters from the bottom right corner and heads in the same direction as in the query clip. Interestingly, this event is detected earlier by *EPG* than by *TPG*, even before the ambulance was visible. This shows that even though *EPG* and *TPG* both integrate audio and video information, *EPG* is more adept at fusing both modalities, likely due to its training with embedding-based pair generation. By positively pairing segments where the ambulance is audible with segments where it is visible, *EPG* improves its ability to identify such events. Furthermore, in two other appearances the ambulance follows a different trajectory, entering from the top left and turning right. Only *EPG* and *TPG* successfully detect these cases. Additionally, there is one other instance where the broadcast audio is present, detected only by *EPG* and MAViL.

In example (C), a police car enters the scene with loud sirens and flashing warning lights. Although the car itself is rather small, its visual and auditory features make it distinct from other vehicles. *EPG*, *TPG*, and MVAiL all locate another similar event. Again, *EPG* detects the event earlier, even before the car is visible. All three methods also successfully identify other occurrences where police cars drive by or sirens are audible in the background. However, the baseline method (*A + V*) is unable to find a matching event for this example. The examples of the ambulance and the police car demonstrate that our proposed embedding based pair generation and custom loss function help to keep more information and cover more aspects of the event, which benefits the real-world application.

Although this qualitative evaluation is limited in scope, it is important to emphasize that these results are derived using the same representations applied in the anomaly detection task. This highlights the versatility and robustness of the learned embeddings across multiple tasks.

### 5.4 Ablation study

To have a better understanding of our method, we analyze the results for different values of the hyperparameters used in our approach, namely, δ,σ , and ω . Since δ adapts based on the similarity of positive pairs, we focus our evaluation on σ and ω , which represent the temporal constraints and the weight between the distance functions, respectively. Since the only annotated task in our experiments is audio-visual event localization, we restrict our ablation study to this task.

#### 5.4.1 Temporal constraint σ

As shown in Table 2, the value of the temporal constraint σ has minimal impact on our approach. Starting from the second epoch, the embedding-based pair mechanism is introduced, which does not solely rely on the time difference but also considers the semantic similarity in selecting pairs. This dual mechanism offers flexibility, as the optimal temporal constraint σ can vary based on the content of the data. For instances, two frames that are 1 min apart in a scene depicting a sidewalk might contain very similar content, whereas the same time gap in a highway scene could result in significantly different content. Given this variability, the robustness of *EPG* shows another advantage in the real-world scenario.

**TABLE 2:** Ablation study on different σ . The impact of different σ on audio-visual event localization results.

σ (sec)	0	1	10	30	60	120
Similar	86.92	86.21	85.32	87.41	87.92	86.31
Challenging	77.48	78.62	77.33	79.82	79.82	78.32

#### 5.4.2 Weight in distance function ω

Table 3 shows the effect of different values for ω , which is used in Equation 5. Both cosine similarity and Euclidean distance are commonly used as distance metrics in contrastive learning (Hadsell et al., 2006; Zeng et al., 2020). When ω is set to 0.25, 0.5, or 0.75, there is no significant impact on the performance of audio-visual event localization. However, when only one of the distance metrics is used (ω=0,ω=1) , we observe that the model sometimes fails to converge. We investigated the learning process and hypothesize why the instability arises when using only Euclidean distance or cosine similarity. On the one hand, as the pre-trained visual encoder $F_{v}$ is not normalized to a unit vector, we did not normalize the output of $F_{a}$ either. In the early stages of the training, using only cosine similarity occasionally leads to the model collapsing or diverging. Cosine similarity does not reflect the magnitude of the vector, which can be a crucial factor in measuring the spatial correlation between two data points in a non-unified embedding space.

**TABLE 3:** Ablation study on different ω . DNC stands for *Did not converge*. The impact of different ω on audio-visual event localization results.

ω	0	0.25	0.5	0.75	1
Similar	DNC	85.21	86.92	85.44	DNC
Challenging	DNC	75.16	77.48	76.83	DNC

In contrast, Euclidean distance provides more efficient guidance in training the $F_{a}$ more efficiently. However, we found that a poor choice of the margin constant also leads to model collapse or divergence when using only Euclidean distance. Since the optimal margin for Euclidean distance can vary depending on the data, incorporating cosine similarity helps balance the loss function, leading to more stable training. Moreover, Euclidean distance complements cosine similarity by considering the absolute difference between two vectors. The combination of both metrics helps in identifying semantically similar events in the embedding space, as it captures both directional and magnitude-based relationships.

## 6 Conclusion and future work

In this paper, we discussed the challenges of learning audio-visual representations from multi-modal surveillance data. We addressed the limitations of relying solely on temporal alignment as pretext task, as well as the minimal sufficient representation bottleneck inherent in contrastive learning. To the best of our knowledge, this is the first study to explore these issues in the context of surveillance data.

We introduced a novel embedding-based pair generation mechanism that mitigates the problem of false negative pair generation while promoting more diversity in positive pairs. Our pseudo-Siamese network, enhanced by a new contrastive loss function that accounts for multiple positive pairs, learns more effective audio-visual representations.

We evaluated the generalization of the learned representations across various downstream tasks and compared our approach to state-of-the-art approaches using a publicly available dataset. Additionally, we demonstrated the effectiveness of our method on a more challenging dataset of real-world surveillance data. Our results show that our approach performs similar or better than existing state-of-the-art techniques.

In future work, we will further explore how the learned representations perform on different downstream tasks. We will also investigate techniques to make the model smaller and faster. Both audio and video data are potentially privacy sensitive and should not leave the local edge device unless absolutely necessary. To facilitate this, we aim to optimize the model for real-time operation on resource-constrained platforms, enabling scalable deployment while preserving privacy.

## Data Availability

The datasets presented in this study can be found in online repositories. The names of the repository/repositories and accession number(s) can be found in the article/supplementary material.

## Footnotes

1
https://github.com/sainathadapa/dcase2019-task5-urban-sound-tagging

2
https://www.youtube.com/watch?v=2gZySUir8_w$

3
https://www.youtube.com/watch?v=xiLF6PmFZP4$
